# Supplementary material for: Functional Characterization of the Cystine-Rich-Receptor-like Kinases (CRKs) and Their Expression Response to Sclerotinia sclerotiorum and Abiotic Stresses in Brassica napus
Source: Int J Mol Sci. 2022 Dec 28;24(1):511. doi: 10.3390/ijms24010511 (PMC9820174; doi:10.3390/ijms24010511)
Supplement: Supplementary file 1 [file ijms-24-00511-s001.zip › Figure S1.pdf]

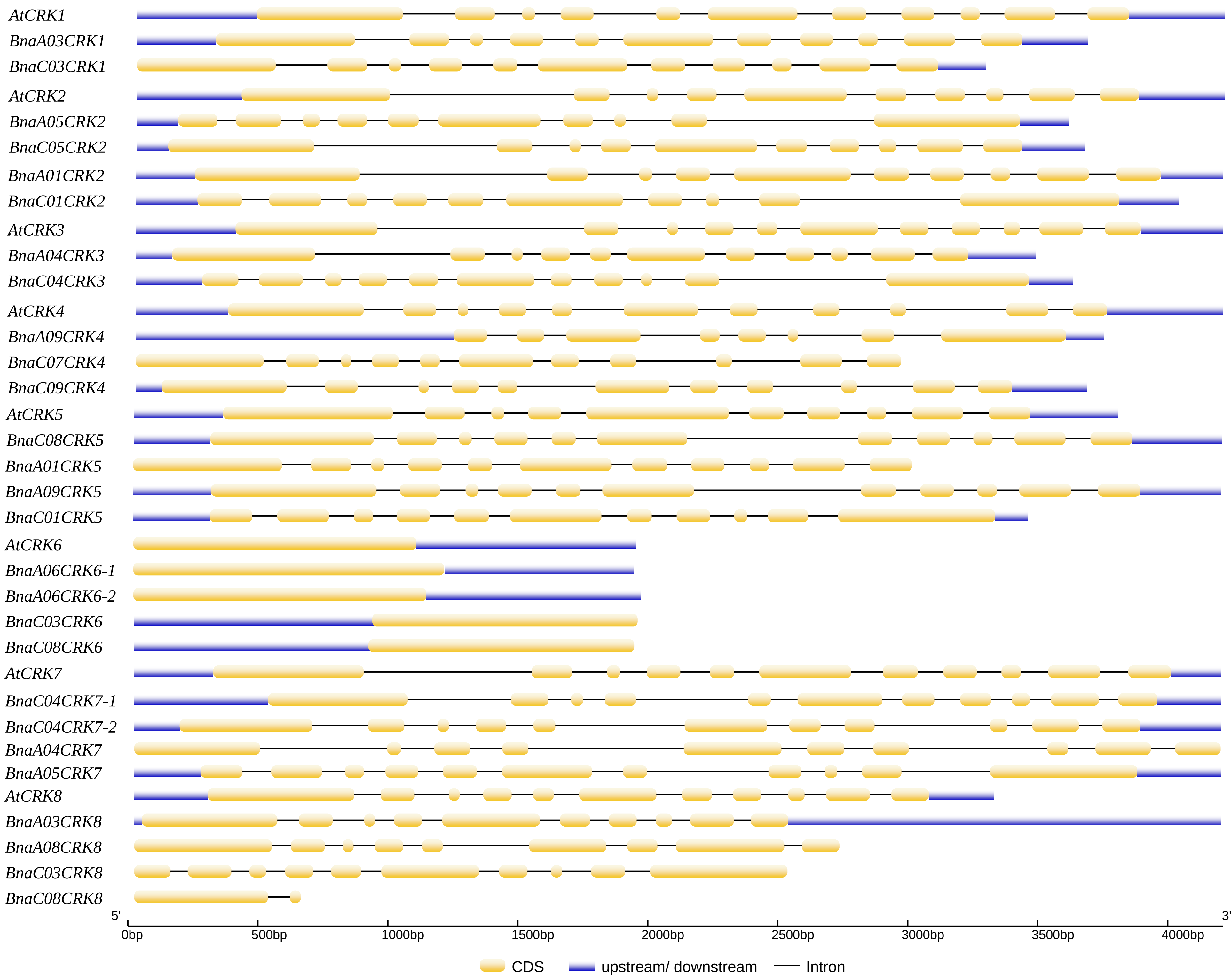

Fig. S1. Distribution of the exon and intron of the *CRK* gene family in *B. napus*. The yellow round block and thin black line indicate exon and intron. The Y-axis displays each group of *BnaCRKs*, while the X-axis represents the length of the exons and introns.
