# Supplementary material for: Functional Characterization of the Cystine-Rich-Receptor-like Kinases (CRKs) and Their Expression Response to Sclerotinia sclerotiorum and Abiotic Stresses in Brassica napus
Source: Int J Mol Sci. 2022 Dec 28;24(1):511. doi: 10.3390/ijms24010511 (PMC9820174; doi:10.3390/ijms24010511)
Supplement: Supplementary file 1 [file ijms-24-00511-s001.zip › Figure S5.pdf]

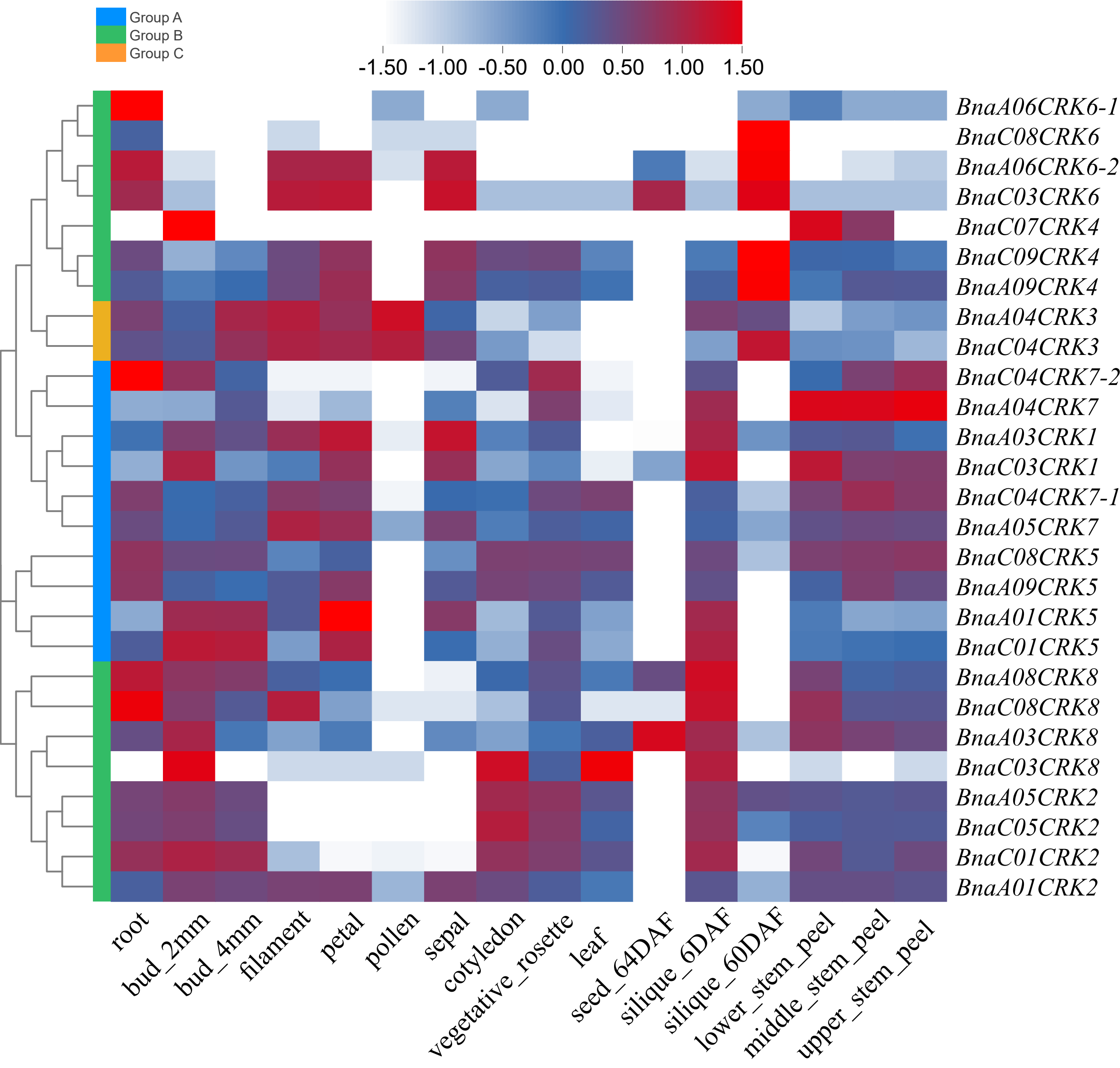

Fig. S5. Heatmap of the *BnaCRKs* expression in root, bud, filament, petal, pollen, sepal, cotyledon, vegetative rosette, leaf, seed, silique, and stem tissues. The different groups of *BnaCRKs* are highlighted in dissimilar colors. The normalized log2 values are listed in Table S7.
