# Supplementary material for: Functional Characterization of the Cystine-Rich-Receptor-like Kinases (CRKs) and Their Expression Response to Sclerotinia sclerotiorum and Abiotic Stresses in Brassica napus
Source: Int J Mol Sci. 2022 Dec 28;24(1):511. doi: 10.3390/ijms24010511 (PMC9820174; doi:10.3390/ijms24010511)
Supplement: Supplementary file 1 [file ijms-24-00511-s001.zip › Figure S6.pdf]

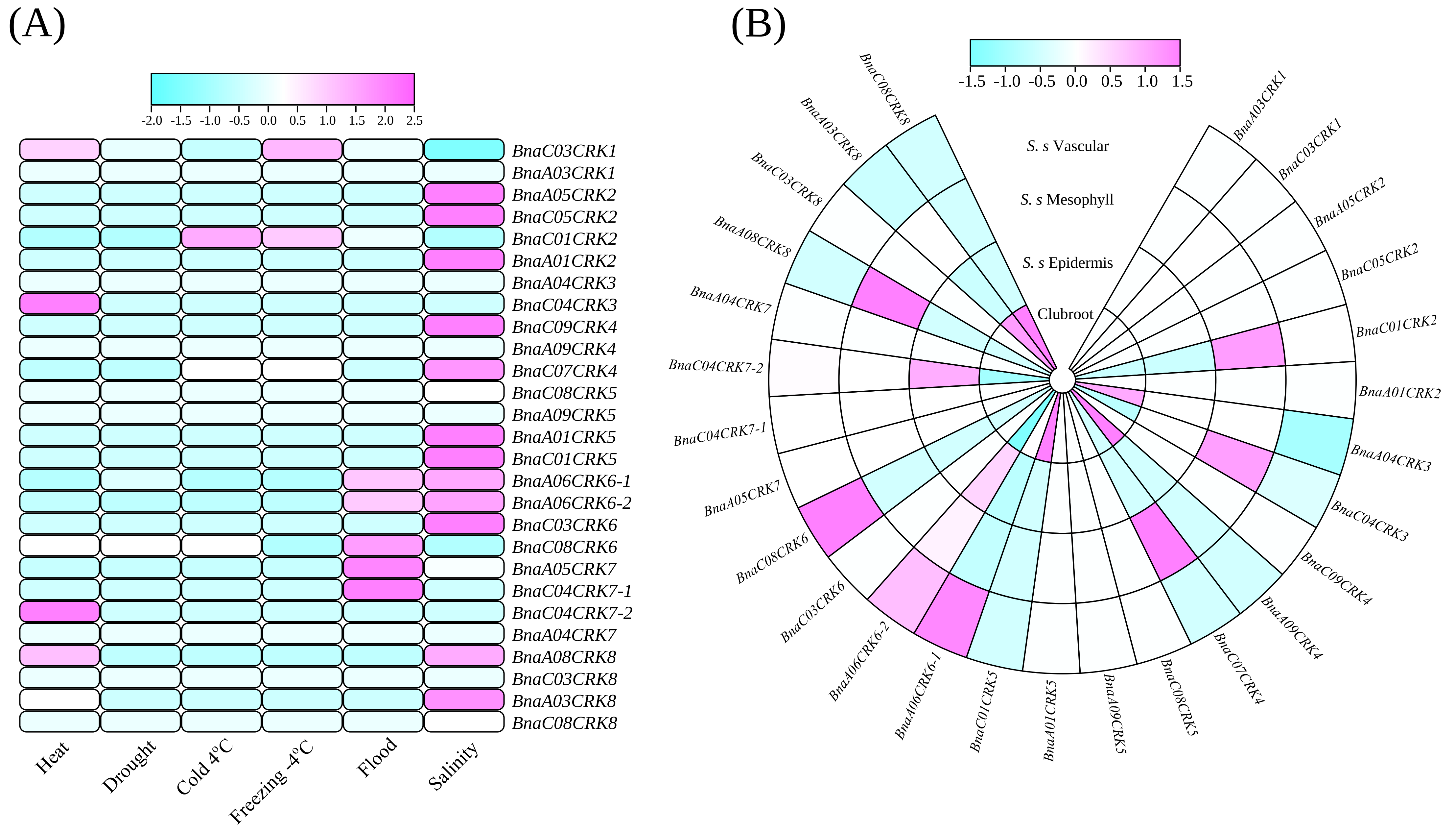

Fig. S6. Transcriptomic data of *BnaCRKs*. **(A)** *BnaCRKs* expression profile in response to heat, drought, cold, freezing, salinity and flood. **(B)** *BnaCRKs* expression profile in response to *S. s* (*Sclerotinia sclerotiorum*) and clubroot. The expression data was normalized by Log2 method and the values ae listed in Table S9.1. The color bar show the expression level ranging from lower to higher.
