# Supplementary material for: Functional Characterization of the Cystine-Rich-Receptor-like Kinases (CRKs) and Their Expression Response to Sclerotinia sclerotiorum and Abiotic Stresses in Brassica napus
Source: Int J Mol Sci. 2022 Dec 28;24(1):511. doi: 10.3390/ijms24010511 (PMC9820174; doi:10.3390/ijms24010511)
Supplement: Supplementary file 1 [file ijms-24-00511-s001.zip › Figure S7.pdf]

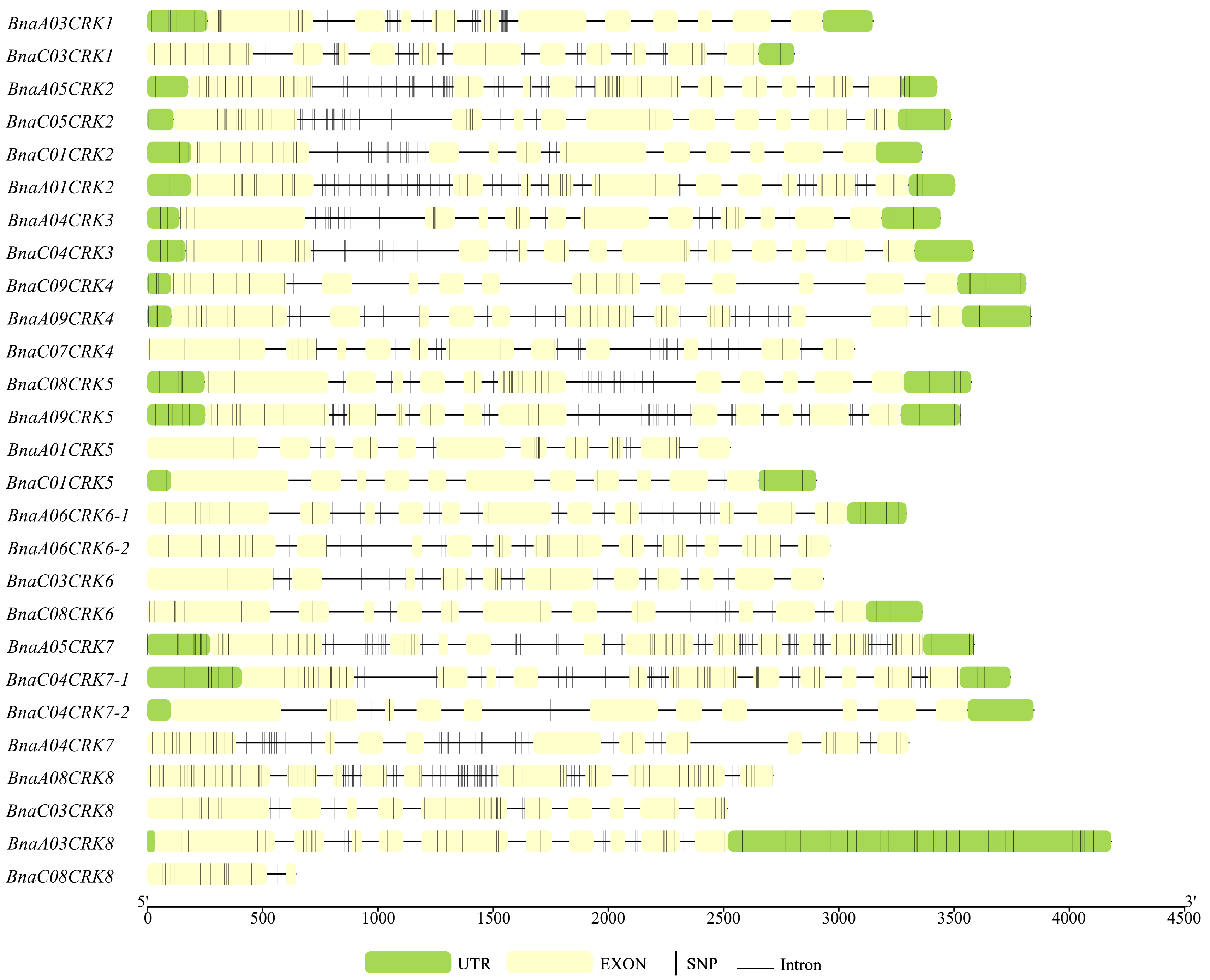

Fig. S7. Single nucleotide polymorphisms (SNPs) frequency on the *BnaCRKs* gene structure. The pale yellow colored block indicates the exon, while the thin black short lines on the top represents the SNPs.
