# Supplementary figures and images for: Functional Characterization of the Cystine-Rich-Receptor-like Kinases (CRKs) and Their Expression Response to Sclerotinia sclerotiorum and Abiotic Stresses in Brassica napus
Source: Int J Mol Sci. 2022 Dec 28;24(1):511. doi: 10.3390/ijms24010511 (PMC9820174; doi:10.3390/ijms24010511)

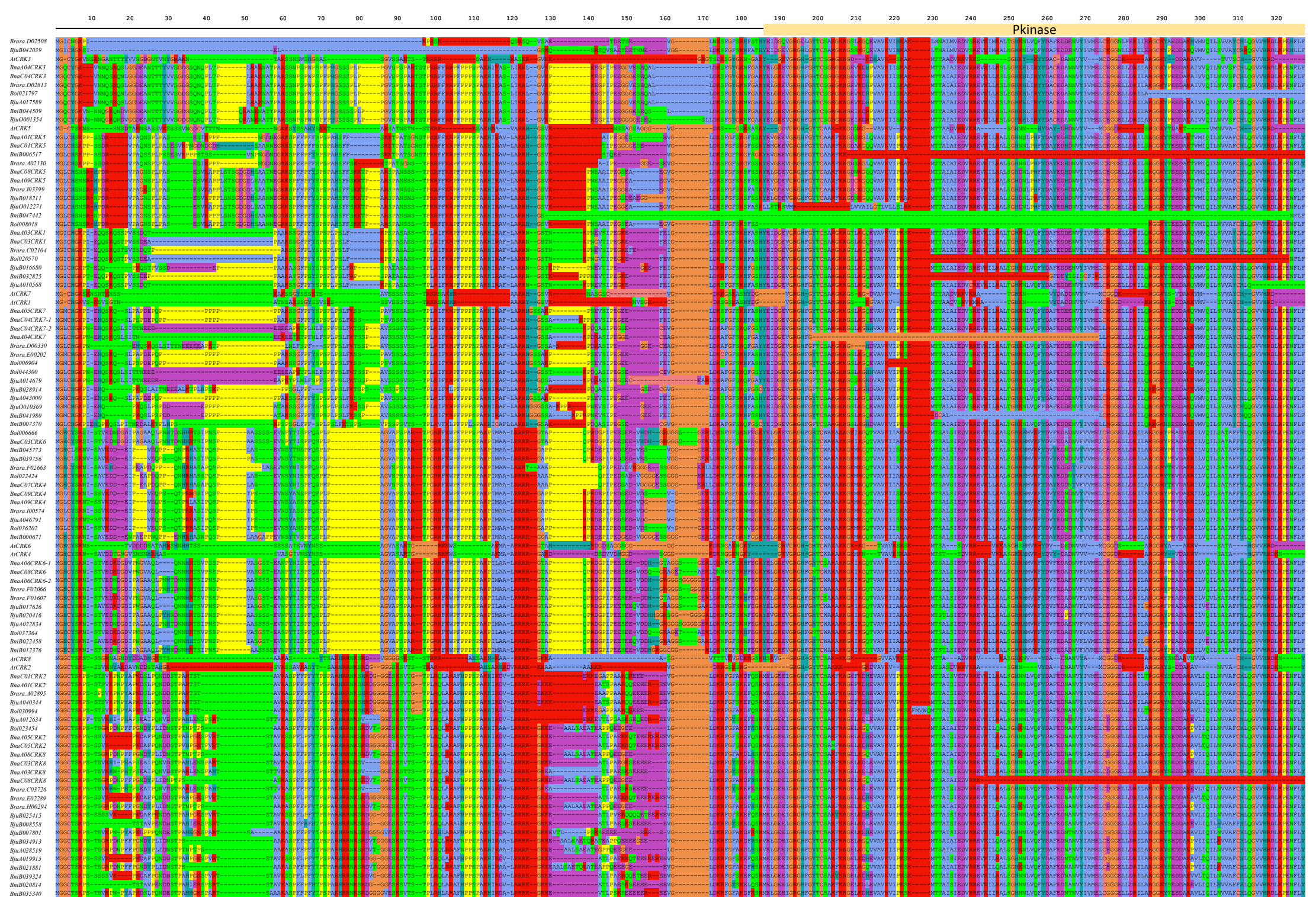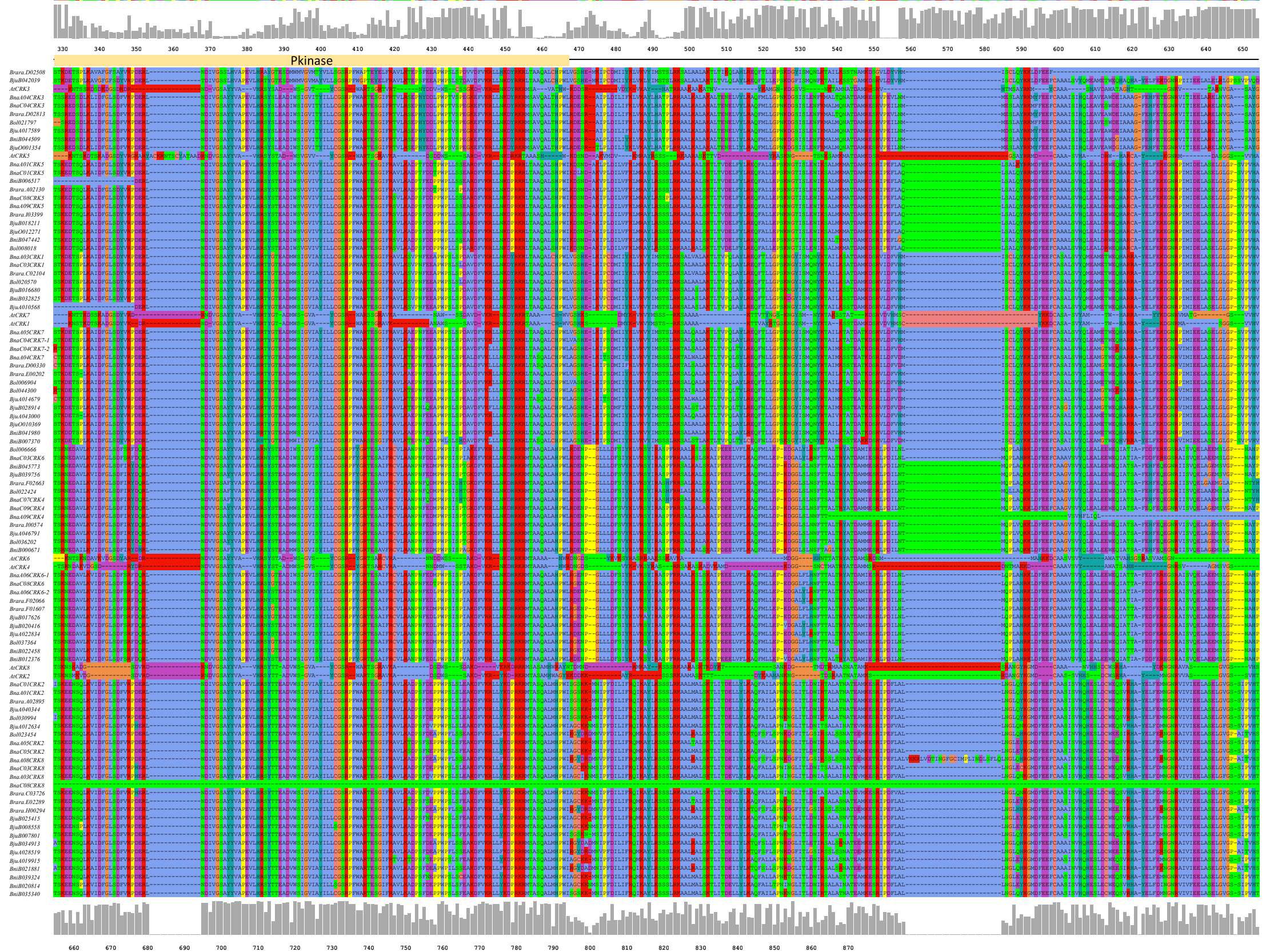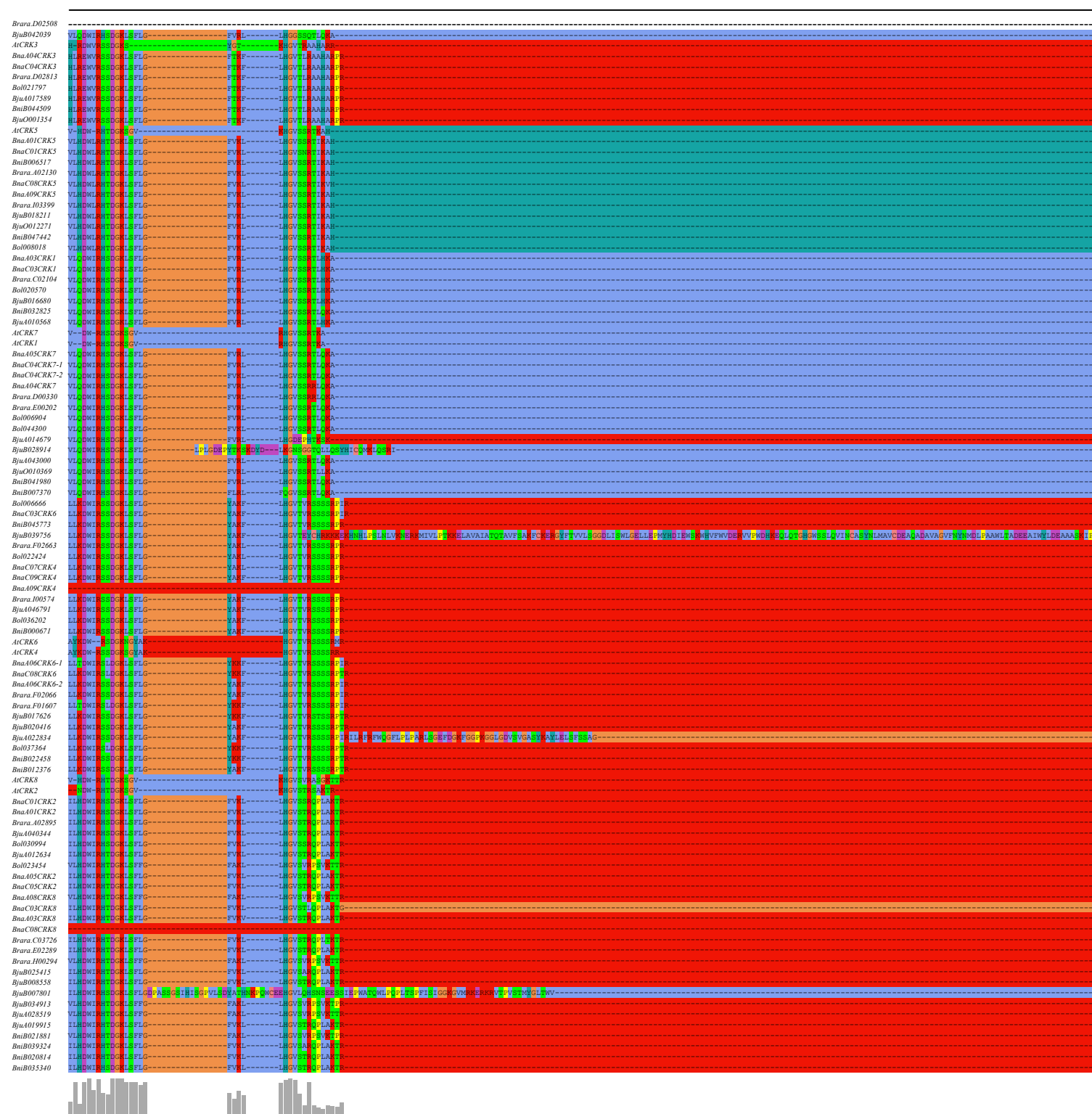

Fig. S2. Multiple sequence alignment of CRK proteins from *Brassicaceae* species

Supplement: Supplementary file 1 [file ijms-24-00511-s001.zip › Figure S2.pdf]

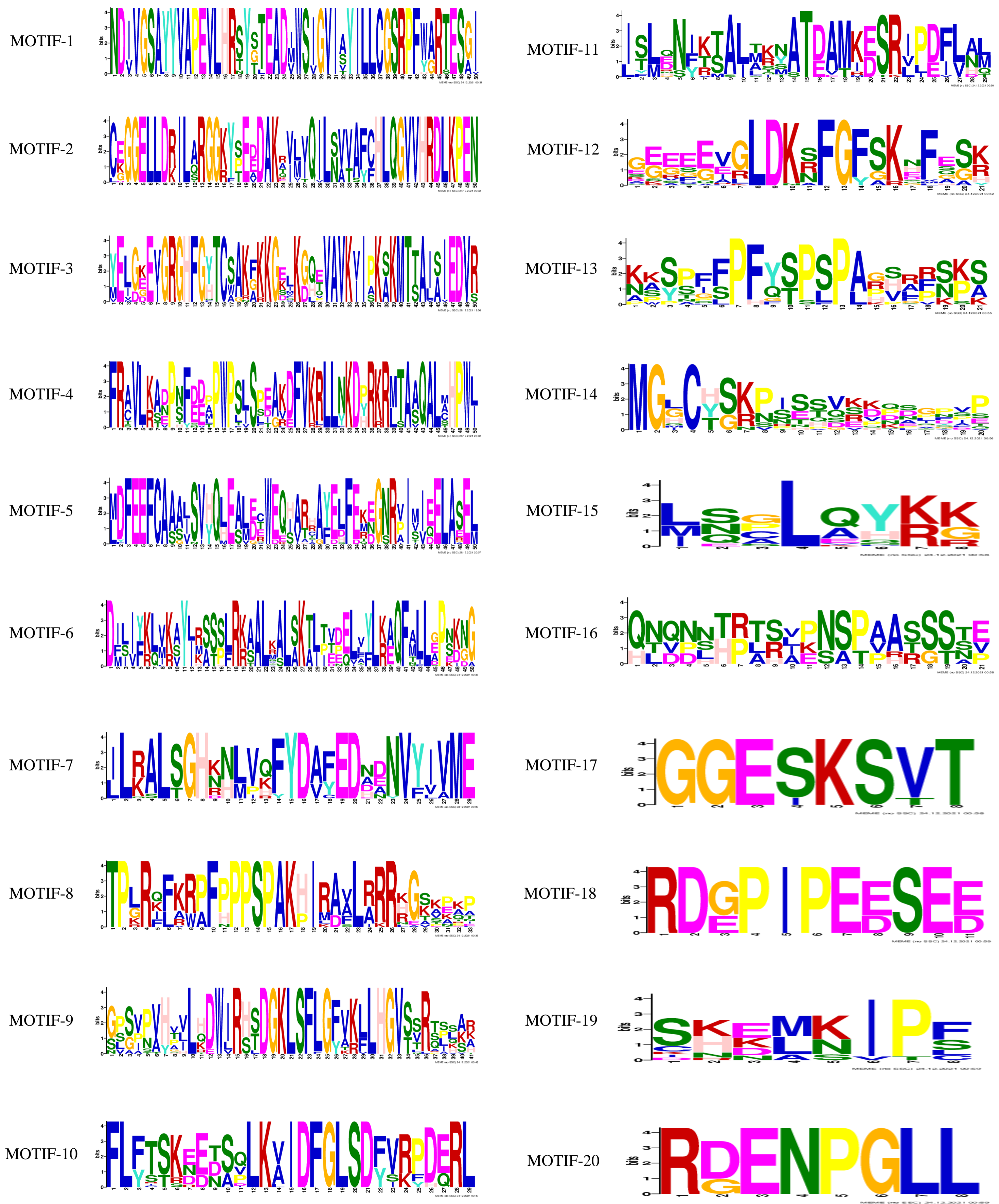

**Fig. S3.** Schematic diagram of *BnCRKs* motifs.

Supplement: Supplementary file 1 [file ijms-24-00511-s001.zip › Figure S3.pdf]
